# Iron Deficiency Anemia and Dyslipidemia Among Hospital Nurses: A Cross-Sectional Study in Turkey

**DOI:** 10.3390/jcm13237042

**Published:** 2024-11-22

**Authors:** Volkan Medeni, Rabia Aygür, İrem Medeni, Kübra Nur Türk, Asiye Uğraş Dikmen, Mustafa Necmi İlhan

**Affiliations:** 1 Department of Public Health, Faculty of Medicine, Gazi University, Ankara 06500, Türkiye; 2 Employee Health Department, General Directorate of Public Health, Ministry of Health, Ankara 06100, Türkiye

**Keywords:** iron deficiency anemia, hyperlipidemias, nurses, hospitals, hematologic tests

## Abstract

**Introduction**: Anemia and dyslipidemia are significant health concerns that affect individual health and societal development. This study aimed to determine the prevalence of iron deficiency anemia and dyslipidemia among nurses in a university hospital in Turkey and explore potential relationships between these conditions. **Methods**: This cross-sectional study was conducted among 712 nurses who underwent periodic health examinations. Data on demographic characteristics, hemoglobin, iron parameters, ferritin, transferrin saturation, and lipid profile were analyzed. People with all four hemoglobin, iron, ferritin, and transferrin saturation values lower than normal ranges at the same time were considered to have iron deficiency anemia. **Results**: Iron deficiency anemia prevalence was 10.7%, with no cases observed in male nurses or those aged 51 and older. Among the nurses, approximately 16.3% had low hemoglobin levels, 16.6% had low hematocrit levels, 30.6% had low ferritin levels, 36.0% had low transferrin saturation, 40.3% had low iron levels, and 24.9% high iron-binding capacity. Elevated total cholesterol was observed in 34.8%, high LDL in 29.6%, low HDL in 27.0%, and elevated triglycerides in 15.0%. Nurses with iron deficiency anemia had significantly lower triglyceride levels than those without. Weak positive correlations were found between triglycerides and hemoglobin, iron, ferritin, and transferrin levels. Additionally, higher total cholesterol, LDL, and triglyceride levels were associated with increased hemoglobin levels. **Conclusions**: This study highlights the high prevalence of iron deficiency anemia and dyslipidemia among hospital nurses, with a notable association between these conditions and factors such as age, gender, and dietary habits. Our findings underscore the need for healthcare services to prioritize the prevention, diagnosis, and management of these health issues in healthcare workers. A comprehensive approach, including regular screenings, dietary improvements, and addressing workplace factors could improve health outcomes and enhance healthcare delivery.

## 1. Introduction

The World Health Organization defines anemia as a reduction in either the number of red blood cells or the concentration of hemoglobin within them, falling below specific threshold values set according to a person’s age, gender, and physiological status [1]. Anemias can be classified based on erythrocyte morphology as microcytic, normocytic, or macrocytic, or according to underlying causes, such as certain malignancies, chronic diseases, genetic factors, and nutritional deficiencies [2]. Anemia is an important disease that threatens health and negatively affects social and economic development in developing and developed countries, affecting a population of 1.92 billion worldwide and causing 52.0 million years of disability [3]. It is estimated that every USD1 investment to prevent anemia can prevent an economic loss of approximately USD12, and this investment has the potential to increase economic productivity by USD110 billion, especially for women and children in low- and middle-income countries [4].

The most common type of anemia is iron deficiency anemia [5]. Iron deficiency anemia may result from a variety of factors, including increased iron requirements during infancy, adolescence, and pregnancy; insufficient dietary iron intake due to poor nutrition; reduced intestinal absorption; chronic blood loss caused by parasitic infections, heavy menstrual periods, or hematuria; and underlying chronic disease [6]. It impacts quality of life across all ages but poses particular challenges in women’s health, contributing to risks like preterm birth, low birth weight, and maternal mortality in reproductive-age women, while also limiting work capacity due to reduced productivity [7].

Unhealthy eating habits and lifestyle disrupt serum lipid metabolism as well as anemia and lead to many health problems, including dyslipidemia, metabolic syndrome, and cardiovascular diseases. Dyslipidemia, which is defined as the absence of cholesterol and lipid parameters measured in serum between normal limits, is one of the important causes of disease burden [8]. The World Health Organization has reported that the prevalence of dyslipidemia is 39%, and high cholesterol leads to 2.6 million deaths (4.5% in total) and 29.7 million disability-adjusted life years (2% in total) [9]. In Turkey, dyslipidemia and anemia are notable public health issues, with about three in ten adults experiencing high cholesterol, one in two with low HDL levels, and one in three facing elevated triglycerides [10]. Many genetic and environmental factors are among the causes of dyslipidemia. Modifiable risk factors include dietary habits and lifestyle [11].

The results of studies investigating the relationship between anemia and cholesterol and triglyceride levels point in different directions [12,13]. Although some experimental studies have found relationships between dietary iron intake and serum lipid and lipoprotein concentrations, such relationships have not been extensively investigated in humans, and the available data need to be more consistent [14,15].

The health status of nurses, who have an essential role in health service delivery and patient care, may affect their work performance and, thus, the quality of the service provided to patients. The nursing profession may affect nutritional habits from time to time due to factors such as intensive working hours and stressful environments. Since this situation increases the risk of iron deficiency anemia, it is necessary to determine the frequency of iron deficiency anemia in nurses and take precautions if necessary. Determining the frequency of dyslipidemia in nurses and making interventions may also be a step in reducing the risk of cardiovascular disease. The fact that the existing data contain contradictory results and that insufficient comprehensive research has been conducted shows the need for further studies in this field. In this study, we aimed to determine the frequency of iron deficiency anemia and dyslipidemia in nurses working in a university hospital and to examine the relationship between these parameters.

## 2. Materials and Methods

The study is cross-sectional type. It was conducted in Gazi Hospital, one of Turkey’s largest tertiary healthcare institutions. Since 1 November 2021, the Employee Health Unit has been serving within the hospital. Here, all employees in the hospital undergo periodic health examinations at least once a year. During these examinations, blood samples are routinely taken; hemograms, biochemistry, and immunology tests are regularly performed. This unit coordinates the follow-up and treatment of detected health problems.

The study population consisted of 1002 nurses working at the hospital between 1 January and 31 December 2022, who completed hemogram and biochemistry tests as part of their periodic health examinations. Our study focused on analyzing the prevalence of anemia and dyslipidemia, along with any relationship between anemia and lipid profile. To reduce confounding factors, we excluded nurses with conditions known to affect lipid profiles, including obesity, diabetes mellitus, liver disease, and renal failure. These exclusions are meant to help eliminate variables that may have influenced the results.

For diabetes mellitus, we used a fasting blood glucose level of 126 mg/dL or higher, or an HbA1c level of ≥6.5% within the last year as exclusion criteria. Obesity was defined as a Body Mass Index (BMI) of ≥30 kg/m^2^. Additionally, nurses diagnosed with thalassemia or hemoglobinopathy were excluded, as these conditions could produce lab results similar to those seen in iron deficiency anemia. Screening for these exclusions involved reviewing medical histories and specific diagnostic tests. Data on dietary history were obtained from anamnesis forms completed during periodic examinations. Among the foods consumed weekly by the participants, meat products and legumes were categorized as iron-rich foods, green leafy vegetables and fruits as iron absorption supporting foods, and dairy products and grains as other foods. Consumption of 3 or more days per week was considered high, and consumption of none or 1–2 days per week was considered low.

In total, 250 nurses were excluded based on these criteria. No sample selection was made, and the aim was to reach 752 nurses after the inclusion and exclusion criteria. The data of 712 nurses who accepted to participate in the study were analyzed within the scope of the study, and the response rate was 94.7%.

Information about the age, gender, and department of the participating nurses was obtained from hospital records. Informed consent was obtained from the participants during the periodic health examination. Hemoglobin, hematocrit, ferritin, iron, iron-binding capacity, transferrin saturation, total cholesterol, HDL-cholesterol, LDL-cholesterol, and triglyceride levels in the blood test results were evaluated. Ethical approval of the study was obtained from the Gazi University Ethics Commission on 5 July 2022 (2022-854).

Whole blood samples were collected in purple-capped EDTA tubes for hemogram tests, and the tube was gently inverted and mixed 5–6 times as soon as the blood was collected to prevent clot formation. Whole blood samples were taken into yellow-capped gel tubes for biochemistry tests and then centrifuged at 1500× *g* for 10 min. Hemoglobin and hematocrit levels were evaluated using the cyanmethemoglobin method on a Beckman Coulter UniCel DxH 800 Analyzer. Iron, iron-binding capacity, serum ferritin, transferrin, total cholesterol, HDL-cholesterol, and triglyceride levels were measured by autoanalyzer. LDL-cholesterol was calculated using the Friedewald formula: [Total cholesterol − (HDL-cholesterol + triglyceride/5)], transferrin saturation: (iron/total iron binding) × 100.

Normal ranges of blood parameters are determined as follows:

- For hemoglobin: 12.0–14.6 g/dL in women and 13.0–16.9 g/dL in men;
- For hematocrit: 36.6–44.0% in women and 40.0–49.4% in men;
- For ferritin: 11–307 ng/mL in women and 23.9–336.2 ng/mL in men;
- For iron: 60–180 μg/dL in women and 70–180 μg/dL in men;
- For iron-binding capacity:155–355 μg/dL in both men and women;
- For transferrin saturation: 15–45% in both men and women;
- For total cholesterol: <200 mg/dL in both men and women;
- For HDL-cholesterol: >50 mg/dL in women and >40 mg/dL in men;
- For LDL-cholesterol: <130 mg/dL in both men and women;
- For triglycerides: <150 mg/dL in both men and women.

According to the results of blood tests, people with all four hemoglobin, iron, ferritin, and transferrin saturation values lower than normal ranges at the same time are considered to have iron deficiency anemia.

Statistical analyses were performed using Statistical Package for Social Science for Windows 25.0 program. Categorical variables were presented as numbers and percentages; continuous variables as mean ± standard deviation, median, minimum, and maximum value. Comparisons between groups was made with Pearson chi-square and Fisher’s exact tests. The Mann–Whitney U test was used to determine differences for continuous variables. Spearman’s correlation test was used to investigate the relationship between lipid parameters and hemoglobin levels. For all analyses, *p* < 0.05 was statistically significant.

## 3. Results

The study involved participants with a mean age of 35.90 ± 8.94 years (min: 23, max: 62). The average hemoglobin level was 13.13 ± 1.38 g/dL, total cholesterol was 189.63 ± 38.78 mg/dL, HDL-cholesterol was 55.86 ± 11.64 mg/dL, LDL-cholesterol was 114.02 ± 32.24 mg/dL, and triglycerides averaged 99.88 ± 60.26 mg/dL.

Participants were categorized by age: 38.2% were 30 years or younger, 29.2% were aged 31–40, and 32.6% were over 40. Women comprised 91.2% of the nurse participants. Regarding their work environment, 44.9% worked in inpatient wards and 20.1% in intensive care units. Among 712 nurses, iron deficiency anemia was observed in 20.5% of those aged 25 and under, with the prevalence decreasing as age increased. Notably, no cases of anemia were found in the group aged 51 and older. The condition was more common in individuals with lower weekly iron-rich food intake (12.9%) than in those with higher intake (6.8%). These differences were statistically significant (*p* = 0.007, *p* = 0.017) Iron deficiency anemia was present in 11.7% of women, while no cases were observed in men. No significant differences were found in anemia rates across the work areas, or weekly consumption of iron absorption-supporting or other foods (*p* > 0.05) (Table 1).

Iron deficiency anemia was present in 10.7% of all participants. Among male participants, no cases of iron deficiency anemia were detected (0.0%), while 11.7% of female participants had this condition. Across all participants, 16.3% had low hemoglobin levels, 16.6% had low hematocrit, 30.6% had low ferritin, 36.0% had low transferrin saturation, 40.3% had low iron levels, and 24.9% had high iron-binding capacity. Additionally, 34.8% of participants had elevated total cholesterol, 29.6% had high LDL-cholesterol, 27.0% had low HDL-cholesterol, and 15.0% had elevated triglycerides (Table 2).

Hemoglobin levels were lower in 17.5% of participants with normal total cholesterol and 14.1% of those with elevated levels. A similar pattern was seen with LDL-cholesterol and triglycerides. Significant differences were observed between hemoglobin levels and total cholesterol, LDL-cholesterol, and triglyceride levels. (*p* = 0.005, *p* = 0.001, and *p* = 0.023) (Table 3).

Among female participants, those with iron deficiency anemia had lower average levels of total cholesterol (185.16 mg/dL vs. 191.03 mg/dL), LDL-cholesterol (110.70 mg/dL vs. 115.08 mg/dL), and triglycerides (86.14 mg/dL vs. 96.04 mg/dL), but higher HDL-cholesterol (57.32 mg/dL vs. 56.88 mg/dL) than those without anemia. The difference in triglyceride levels was statistically significant (*p* = 0.011) (Table 4).

Supplementary Figure S1 shows weak positive correlations between triglyceride levels and hemoglobin (r = 0.203), along with very weak positive correlations between triglycerides and ferritin (r = 0.175), iron (r = 0.115), and transferrin (r = 0.094). The correlations between total cholesterol and blood parameters are depicted in Supplementary Figure S2, while scatter plots for LDL-cholesterol are presented in Supplementary Figure S3, showing very weak positive correlations with hemoglobin (r = 0.076 for total cholesterol and r = 0.087 for LDL-cholesterol). Supplementary Figure S4 illustrates the association between HDL-cholesterol and blood parameters, with HDL values showing very weak negative correlations with hemoglobin (r = −0.174) and ferritin (r = −0.155). The correlations between lipid profile and blood parameters are shown in Table 5.

## 4. Discussion

In this cross-sectional study, we examined the relationship between iron deficiency anemia and dyslipidemia in nurses working in a hospital in Turkey where periodic health examination services are provided to all employees. Our findings offer valuable insights for clinical practice.

Iron deficiency anemia was identified in approximately one out of ten participants in our study. Globally, it is estimated that one-fourth of the population in Turkey suffers from anemia, with two-thirds of these cases being iron deficiency anemia [16]. Similarly, worldwide statistics indicate that one in three women is affected by iron deficiency anemia [17]. In contrast, our study reported a lower frequency of iron deficiency anemia compared to both global and national averages. This discrepancy could be attributed to the participants’ familiarity with the diagnosis and treatment of iron deficiency anemia, given their profession as nurses. Another contributing factor might be the “healthy worker effect”, which suggests that employees tend to be healthier than the general population. A study in Switzerland found iron deficiency anemia in one out of every six healthcare workers during routine health checks [18]. Comparable results were observed in a study from Thailand [19]. The lower prevalence observed in our study, compared to similar studies, could also result from the treatment administered to those diagnosed with iron deficiency anemia during previous health examinations. The Swiss study noted that participants who were suspected of having iron deficiency anemia were more likely to join the study. This could explain why the frequency of anemia was potentially higher in their study compared to ours.

In our study, one-sixth of the hospital nurses had low hemoglobin and hematocrit levels, conditions directly linked to anemia. Anemia can cause fatigue, weakness, and decreased cognitive function, significantly impacting overall health and work performance. Nurses often work long shifts, manage high-stress situations, and perform physically demanding tasks, all of which can be adversely affected by low hemoglobin levels. Previous studies have highlighted the negative effects of anemia on work capacity and productivity [20,21,22], such as reduced physical endurance, impaired concentration, and increased likelihood of errors, which can compromise the quality of care provided by nurses. A study on healthcare workers found that those with anemia reported higher levels of fatigue and were less able to perform their duties efficiently, leading to increased absenteeism and decreased job satisfaction [23]. Addressing low hemoglobin in nurses is crucial for their health and maintaining the quality of patient care. Ensuring that nurses are healthy and able to perform their duties effectively is essential for the overall functioning of healthcare systems. The demanding nature of the nursing profession means that low hemoglobin levels can lead to increased fatigue, decreased productivity, and a higher risk of burnout. Therefore, it is essential to recognize and address low hemoglobin among nurses to ensure they can provide high-quality care while maintaining their well-being.

In our study, iron deficiency anemia was more common among individuals with a lower weekly intake of iron-rich foods compared to those with a higher intake. A study conducted in the United Kingdom found that participants who reported consuming red meat at least three times per week had a lower risk of iron deficiency anemia compared to those with less frequent consumption of unprocessed red meat [24]. Another study from the United States showed that the decrease in average dietary iron intake over the years increased the risk of iron deficiency anemia [25]. Similarly, a study in France, which aimed to determine a weekly red meat intake level that would not pose a risk for colon cancer, suggested that increasing red meat consumption within specific limits could help prevent iron deficiency anemia [26]. Our findings align with these studies, underscoring the importance of iron-rich dietary habits in reducing the prevalence of iron deficiency anemia. Further research on culturally tailored dietary recommendations could be beneficial for improving iron intake in various populations.

In this study, iron deficiency anemia was observed in one-fifth of participants aged 25 years and younger. The prevalence decreased with advancing age, and notably, no cases of anemia were found among the 40 individuals aged 51 years and older. Our findings align with previous studies examining the relationship between iron deficiency anemia prevalence and age. For instance, a study based on data from the Global Burden of Disease Study observed that anemia prevalence decreases with age from birth, spikes in the 15–24 age group, and reaches its lowest levels between ages 45–64 [27]. A case-control study from Korea reported that while anemia prevalence exceeded 30% among women aged 30–39 and 40–49, it gradually declined, with a significant drop below 10% among women aged 50–59 [28]. Similarly, a study in Cameroon found that anemia prevalence was significantly higher in women under 40 and lower in those aged 50–64 [29]. For women, iron requirements are higher during the reproductive years due to menstruation, which often contributes to a higher prevalence of anemia in younger age groups. However, after menopause, the cessation of menstrual blood loss reduces iron demand, potentially offering a protective effect against anemia. These findings underscore the importance of targeted anemia screening and preventive health services for young female nurses.

Our study revealed that one in three nurses had elevated total cholesterol levels. In comparison, a study on primary healthcare workers in Saudi Arabia found that two out of five participants had elevated total cholesterol levels [30]. The higher prevalence in the Saudi Arabian study might be attributed to the inclusion of more male participants and the lack of exclusion criteria for obese individuals. Another study conducted on female nurses in public and private hospitals in India reported that 42% of participants had total cholesterol levels ≥200 mg/dL [31]. In Nigeria, a study on metabolic syndrome among healthcare workers revealed that 65.7% had elevated total cholesterol, compared to 39.2% in the general population [32]. Against these, in Iran, a study examining the link between shift work and hypercholesterolemia among nurses found hypercholesterolemia in one-fifth of shift workers, compared to one-fifteenth of non-shift workers [33]. It is crucial to recognize that the prevalence of elevated total cholesterol varies across countries due to lifestyle differences, awareness levels, dietary habits, genetic predispositions, preventive health services, and healthcare access. This disparity may stem from the demanding nature of healthcare work, which often includes irregular shifts, high stress, and a fast-paced environment, potentially hindering the ability to maintain a healthy and balanced diet. These findings highlight the need for targeted interventions to address the unique health challenges faced by healthcare professionals.

Our study revealed that mean triglyceride levels were significantly lower in nurses with iron deficiency anemia (86.14 mg/dL) compared to those without iron deficiency anemia (101.52 mg/dL). This aligns with a similar study in Egypt, where the mean serum triglyceride level was 85.67 mg/dL in the iron deficiency anemia group and 107.89 mg/dL in the control group [34]. In Korea, patients with severe iron deficiency (Hb < 8.0 g/dL) exhibited significantly low triglyceride levels, which normalized following iron supplementation [35]. A study from India reported a mean serum triglyceride level of 89.4 mg/dL in patients with anemia, compared to 111.4 mg/dL in the control group, showing lower lipid values, including triglycerides, among those with anemia [36]. However, conflicting findings exist: another Indian study found higher mean triglyceride levels in patients with iron deficiency anemia (151.87 mg/dL) compared to a healthy group (109.99 mg/dL) [37]. Similarly, a Turkish study reported elevated triglyceride and total cholesterol levels in patients with iron deficiency anemia [38]. Iron can affect lipid metabolism in different ways. A high-iron diet may raise triglyceride levels by reducing lipoprotein lipase activity, while iron deficiency may lower triglyceride levels by decreasing the activity of enzymes involved in fatty acid synthesis. However, iron deficiency can also increase triglyceride levels by upregulating lipogenic gene expression and disrupting carnitine biosynthesis. The contradictory results suggest that multiple mechanisms might influence triglyceride levels in the context of iron deficiency. To enhance the clarity and impact of our study, we recognize the necessity of constructing a coherent narrative that integrates these diverse findings. Focusing on the underlying mechanisms by which iron deficiency anemia affects triglyceride metabolism in further studies will provide a clearer, more compelling story.

In this study, the participants with high total cholesterol, LDL-cholesterol, and triglyceride levels had higher hemoglobin levels than healthy participants. In addition, a positive correlation was observed between triglycerides and hemoglobin, iron, ferritin, and transferrin levels. A similar study conducted in Turkey found a moderate to weak correlation between total cholesterol levels and hemoglobin, hematocrit, serum iron, serum ferritin, and transferrin saturation [39]. Studies conducted in Iran and the United States found a weak correlation between hemoglobin, iron, and total cholesterol levels [40,41]. Again, a cohort study conducted in the United States showed that triglyceride and LDL-cholesterol levels were higher in those with higher hemoglobin levels than others [42]. The observed elevation in hemoglobin levels among participants with high total cholesterol, LDL-cholesterol, and triglyceride levels could be attributed to several potential mechanisms. Changes in lipid metabolism may stimulate erythropoiesis by increasing the body’s need for oxygen transport, resulting in a compensatory rise in hemoglobin levels. Additionally, lipid imbalances might indirectly influence iron metabolism, which can affect hemoglobin synthesis. Further research is needed to explore these mechanisms in detail and clarify how lipid levels and hemoglobin interact within the context of overall metabolic health.

Our study has some limitations. Firstly, it was conducted at a single center, and a few individuals did not participate due to reasons such as workload, annual leave, sick leave, or concerns about confidentiality. Therefore, caution should be exercised when generalizing the results to the broader population. Nonetheless, our findings are significant, given the high number of participants involved. As a cross-sectional study, we cannot establish a causal relationship between iron deficiency anemia and dyslipidemia. Longitudinal and randomized controlled studies are necessary for a more comprehensive understanding of these relationships. However, our results provide valuable insights into the associations between the variables examined, contributing to the existing literature on iron deficiency anemia. Importantly, when interpreting our results, one should consider the statistical significance of the correlations observed. Although we identified statistically significant correlations between various parameters, the correlation coefficients were relatively low. This suggests a weak relationship between these variables. Consequently, our findings should be interpreted with caution, as other factors likely contribute to variations in iron metabolism.

## 5. Conclusions

Our findings offer valuable insights into the prevalence and relationship between iron deficiency anemia and dyslipidemia among hospital nurses in Turkey.

Approximately one in ten nurses is affected by iron deficiency anemia, with a higher prevalence observed in women, younger individuals and those with lower weekly consumption of iron-rich foods. These findings highlight the importance of age, gender, and nutritional habits, particularly iron intake, in the prevalence of iron deficiency anemia and suggest that a holistic approach, including dietary improvements and regular screening, is essential for effective management.

Dyslipidemia, which is characterized by elevated total cholesterol and low HDL-cholesterol levels, is also prevalent among the hospital nurses. Although the correlations observed between lipid parameters and various iron metabolism markers were statistically significant, they were weak. This suggests that while an association exists between iron metabolism and lipid profiles, the underlying mechanisms are complex and not yet fully understood.

The importance of occupational health services in both iron deficiency anemia and dyslipidemia is evident, emphasizing the need for targeted preventive measures. The causes of these conditions in healthcare workers should be carefully examined and targeted preventive measures implemented. Supporting nurses’ health through regular screenings, alongside improving workplace factors such as nutrition, physical activity, stress management, and shift schedules, can contribute to better health outcomes and enhance healthcare delivery.

## Figures and Tables

**Table 1:** Sociodemographic characteristics of the participants.

			Iron Deficiency Anemia			
	(*n*)	(%)	Yes		No	
			(*n*)	(%)	(*n*)	(%)
Age Groups (*n* = 712)						
25 years and below	78	11.0	16	20.5	62	79.5
26–30 years old	194	27.2	14	7.2	180	92.8
31–35 years old	88	12.3	9	10.2	79	89.8
36–40 years old	120	16.9	15	12.5	105	87.5
41–45 years old	114	16.0	16	14.0	98	86.0
46–50 years old	78	11.0	6	7.7	72	92.3
51 years and older	40	5.6	0	0.0	40	100.0
			χ^2^ = 17.647		***p* = 0.007**	
Gender (*n* = 712)						
Female	649	91.2	76	11.7	573	88.3
Male	63	8.8	0	0.0	63	100.0
			*NA*			
Department (*n* = 712)						
Ward	320	44.9	37	11.6	283	88.4
Intensive care	143	20.1	17	11.9	126	88.1
Operation room	58	8.2	6	10.3	52	89.7
Outpatient clinic	53	7.4	5	9.4	48	90.6
Emergency room	37	5.2	4	10.8	33	89.2
Ambulatory treatment unit	34	4.8	2	5.9	32	94.1
Imaging unit	30	4.2	4	13.3	26	86.7
Administrative unit	26	3.7	1	3.8	25	96.2
Blood collection unit	11	1.5	0	0.0	11	100.0
			χ^2^ = 4.206		*p* = 0.838	
Weekly Consumption of Iron-Rich Foods						
High	262	36.8	18	6.8	244	93.2
Low	450	63.2	58	12.9	392	87.1
			χ^2^ = 5.676		***p* = 0.017**	
Weekly Consumption of Iron Absorption-Supporting Foods						
High	330	46.3	34	10.3	296	89.7
Low	382	53.7	42	11.0	340	89.0
			χ^2^ = 0.089		*p* = 0.765	
Weekly Consumption of Other Foods						
High	349	49.1	39	11.1	310	88.9
Low	363	50.9	37	10.2	326	89.8
			χ^2^ = 0.178		*p* = 0.671	

**Table 2:** Blood parameters of the participants.

	Male		Female		Total	
	(*n*)	(%)	(*n*)	(%)	(*n*)	(%)
Iron Deficiency Anemia (*n* = 712)						
Yes	0	0.0	76	11.7	76	10.7
No	63	100.0	573	88.3	636	89.3
Hemoglobin (*n* = 712)						
Low	0	0.0	116	17.9	116	16.3
Normal	63	100.0	506	78.0	569	79.9
High	0	0.0	27	4.2	27	3.8
Hematocrit (*n* = 712)						
Low	0	0.0	118	18.2	118	16.6
Normal	58	92.1	510	78.6	568	79.8
High	5	7.9	21	3.2	26	3.7
Ferritin (*n* = 712)						
Low	3	4.8	215	33.1	218	30.6
Normal	60	95.2	434	66.9	494	69.4
Transferrin Saturation (*n* = 712)						
Low	5	7.9	251	38.7	256	36.0
Normal	58	92.1	391	60.2	449	63.0
High	0	0.0	7	1.1	7	1.0
Iron (*n* = 712)						
Low	16	25.4	271	41.8	287	40.3
Normal	47	74.6	373	57.5	420	59.0
High	0	0.0	5	0.8	5	0.7
Iron-Binding Capacity (*n* = 712)						
Low	0	0.0	7	1.1	7	1.0
Normal	59	93.7	469	72.3	528	74.2
High	4	6.3	173	26.7	177	24.9
Total Cholesterol (*n* = 712)						
Normal	46	73.0	418	64.4	464	65.2
High	17	27.0	231	35.6	248	34.8
Triglyceride (*n* = 712)						
Normal	36	57.1	569	87.7	605	85.0
High	27	42.9	80	12.3	107	15.0
HDL-Cholesterol (*n* = 712)						
Low	16	25.4	176	27.1	192	27.0
Normal	47	74.6	473	72.9	520	73.0
LDL-Cholesterol (*n* = 712)						
Normal	47	74.6	454	70.0	501	70.4
High	16	25.4	195	30.0	211	29.6

**Table 3:** Relationship between hemoglobin levels and lipid profile of the participants.

	Hemoglobin Levels					
	Low		Normal		High	
	(*n*)	(%)	(*n*)	(%)	(*n*)	(%)
Total Cholesterol (*n* = 712)						
Normal	81	17.5	373	80.4	10	2.2
High	35	14.1	196	79.0	17	6.9
	χ^2^ = 10.560		***p* = 0.005**			
LDL-Cholesterol (*n* = 712)						
Normal	88	17.6	402	80.2	11	2.2
High	28	13.3	167	79.1	16	7.6
	χ^2^ = 13.060		***p* = 0.001**			
HDL-Cholesterol (*n* = 712)						
Normal	81	15.6	421	81.0	18	3.5
Low	35	18.2	148	77.1	9	4.7
	χ^2^ = 1.425		*p* = 0.490			
Triglyceride (*n* = 712)						
Normal	101	16.7	486	80.3	18	3.0
Low	15	14.0	83	77.6	9	8.4
	χ^2^ = 7.571		***p* = 0.023**			

**Table 4:** Relationship between iron deficiency anemia and lipid profile of female participants.

	Iron Deficiency Anemia	
	Yes	No
Total Cholesterol (mg/dL)		
Mean ± SD	185.16 ± 35.45	191.03 ± 39.08
Median	182.00	186.00
Range (min–max)	115.00–320.00	104.00–329.00
	*p* = 0.231	
HDL-Cholesterol (mg/dL)		
Mean ± SD	57.32 ± 11.94	56.88 ± 11.17
Median	55.00	55.00
Range (min–max)	38.00–93.00	32.00–110.00
	*p* = 0.957	
LDL-Cholesterol (mg/dL)		
Mean ± SD	110.70 ± 30.05	115.08 ± 32.49
Median	109.00	112.00
Range (min–max)	48.00–191.00	35.00–229.00
	*p* = 0.308	
Triglyceride (mg/dL)		
Mean ± SD	86.14 ± 49.48	96.04 ± 53.89
Median	70.50	81.00
Range (min–max)	24.00–280.00	27.00–418.00
	***p* = 0.044**	

**Table 5:** Correlations between lipid profile and blood parameters of all participants.

	Hemoglobin		Ferritin		Iron		Transferrin	
	r	*p*	r	*p*	r	*p*	r	*p*
Total Cholesterol	0.076	**0.043**	0.047	0.206	0.051	0.171	0.012	0.749
LDL-Cholesterol	0.087	**0.020**	0.048	0.203	0.017	0.654	−0.010	0.781
HDL-Cholesterol	−0.174	**<0.001**	−0.155	**<0.001**	0.049	0.190	0.018	0.629
Triglyceride	0.203	**<0.001**	0.175	**<0.001**	0.115	**0.002**	0.094	**0.012**

## Data Availability

Data and materials are available on request to authors.

## Supplementary Materials

The following supporting information can be downloaded at: https://www.mdpi.com/article/10.3390/jcm13237042/s1; Figure S1; Scatter plots showing the relationship between triglyceride and blood parameters; Figure S2; Scatter plots showing the relationship between total cholesterol and blood parameters; Figure S3; Scatter plots showing the relationship between LDL-cholesterol and blood parameters; Figure S4; Scatter plots showing the relationship between HDL-cholesterol and blood parameters.
