# Supplementary material for: Iron Deficiency Anemia and Dyslipidemia Among Hospital Nurses: A Cross-Sectional Study in Turkey
Source: J Clin Med. 2024 Nov 22;13(23):7042. doi: 10.3390/jcm13237042 (PMC11642562; doi:10.3390/jcm13237042)
Supplement: Supplementary file 1 [file jcm-13-07042-s001.zip › jcm-3250838-supplementary.pdf]

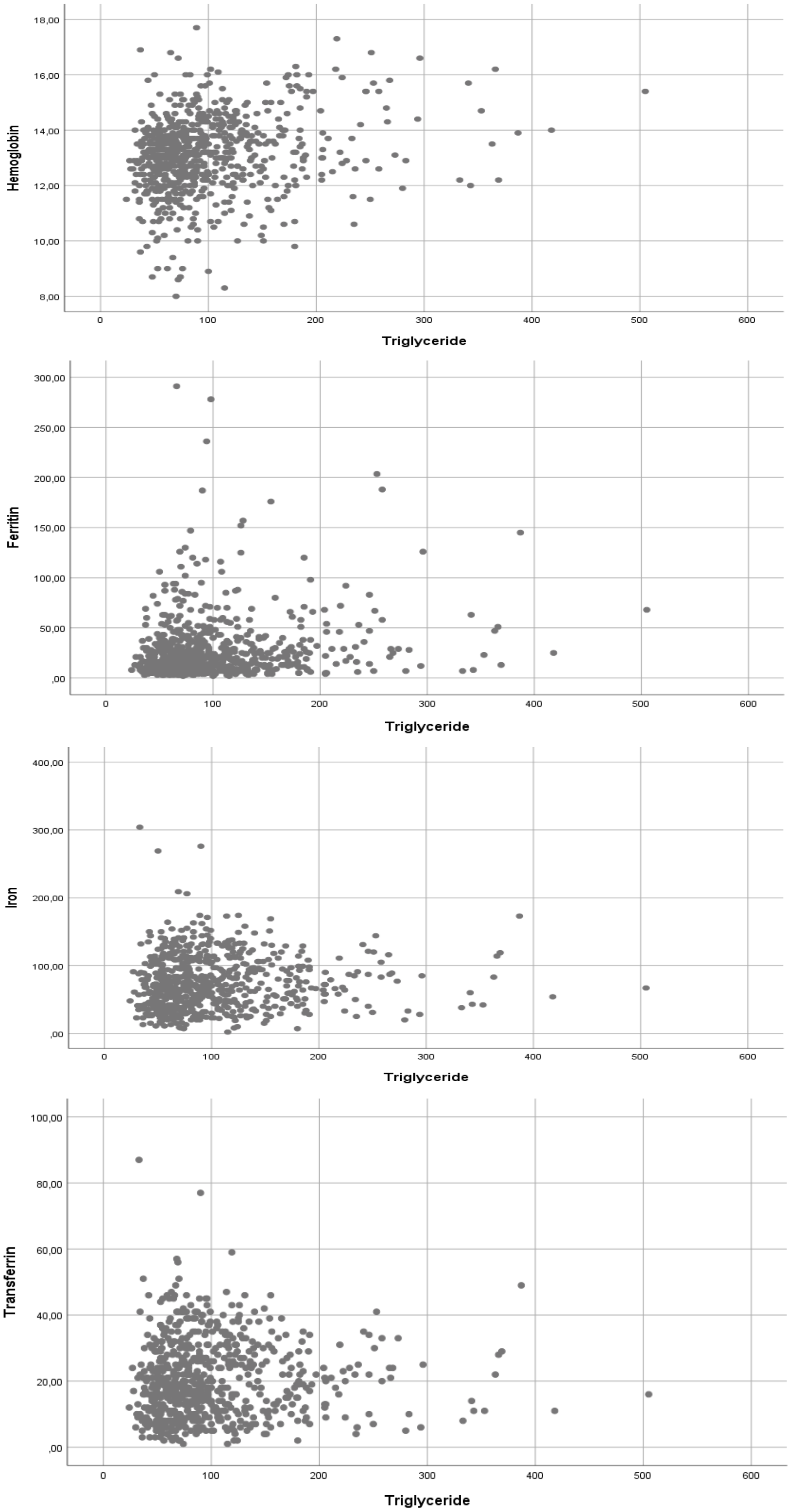

Supplementary Figure S1. **Scatter plots showing the relationship betweentriglyceride and blood parameters**

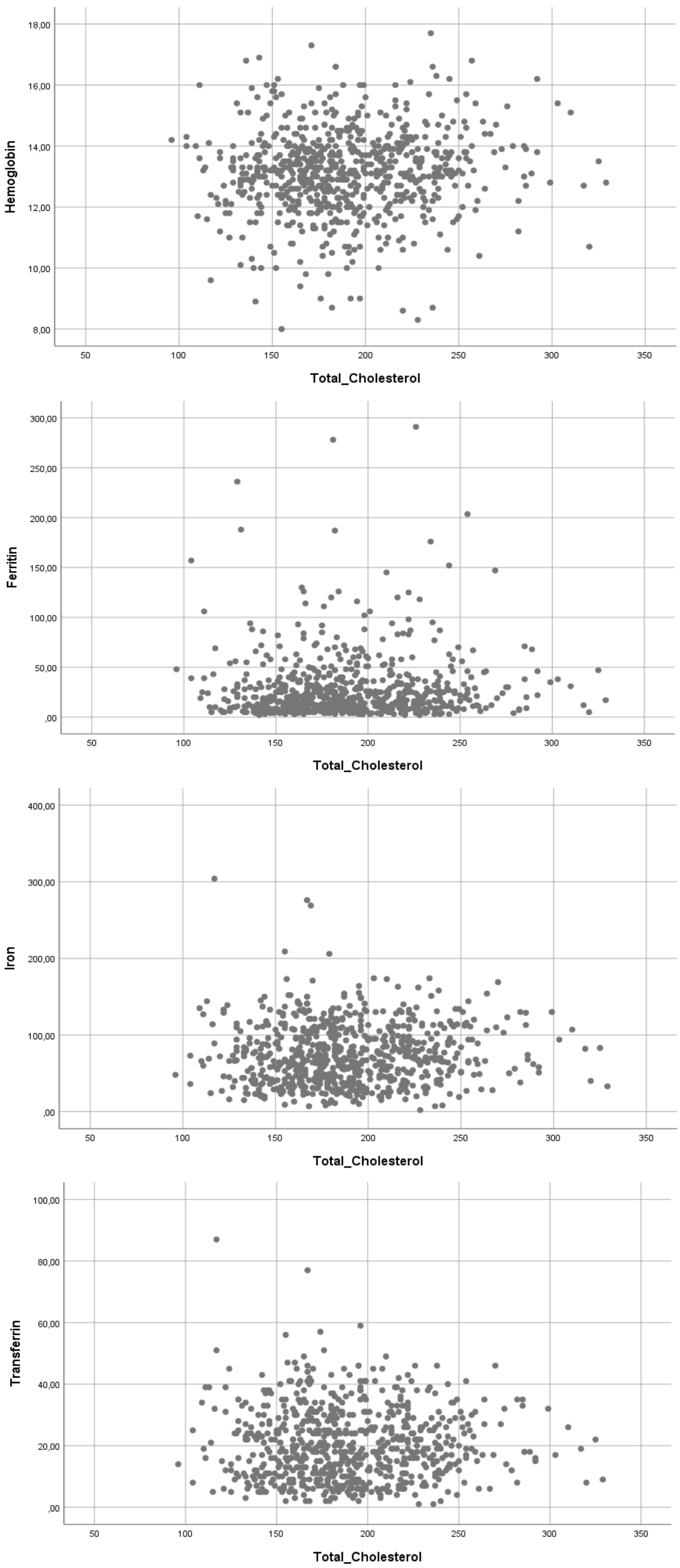

Supplementary Figure S2. Scatter plots showing the relationship between total cholesterol and blood parameters

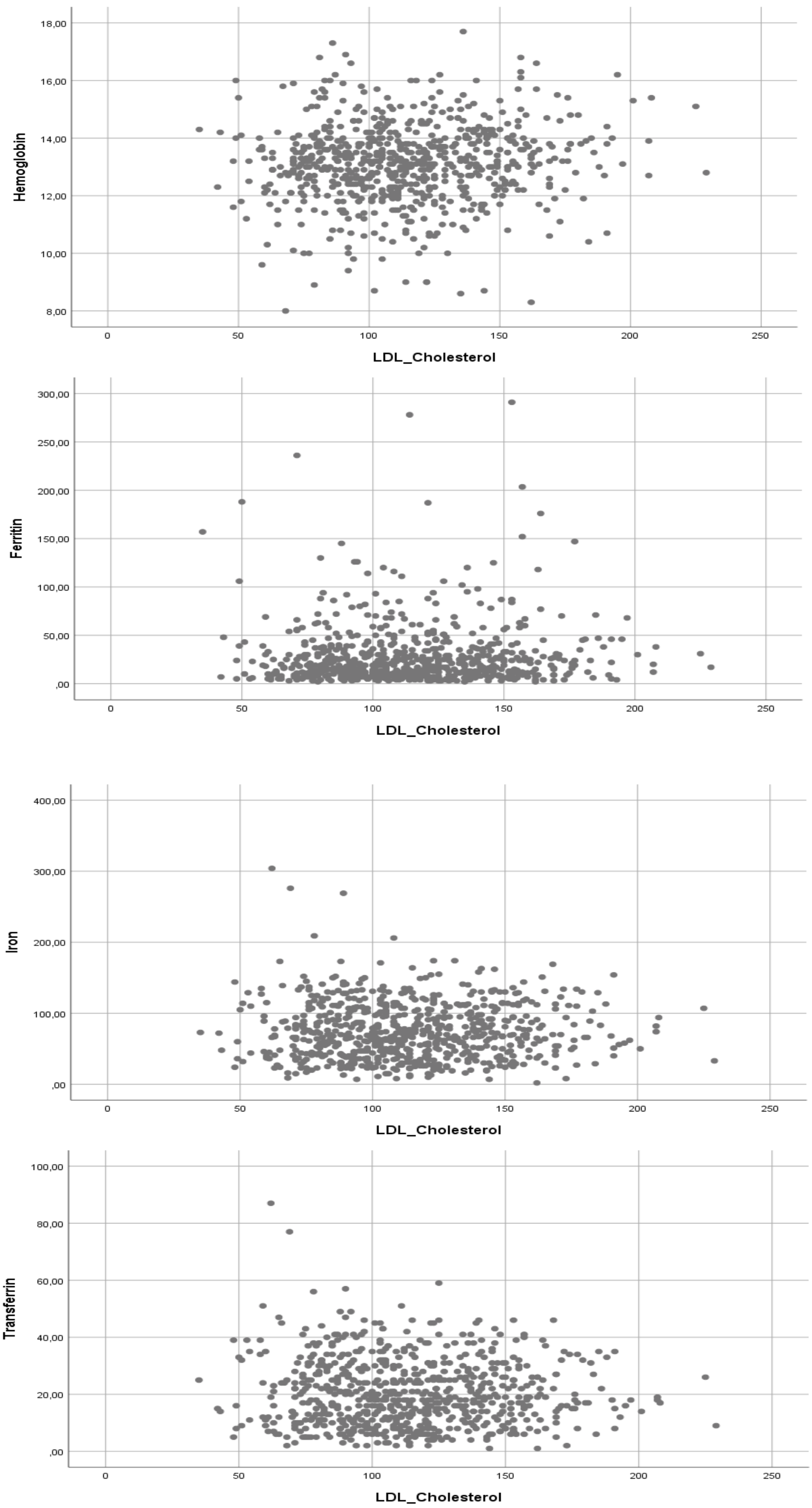

Supplementary Figure S3. **Scatter plots showing the relationship between LDL-cholesterol and blood parameters**

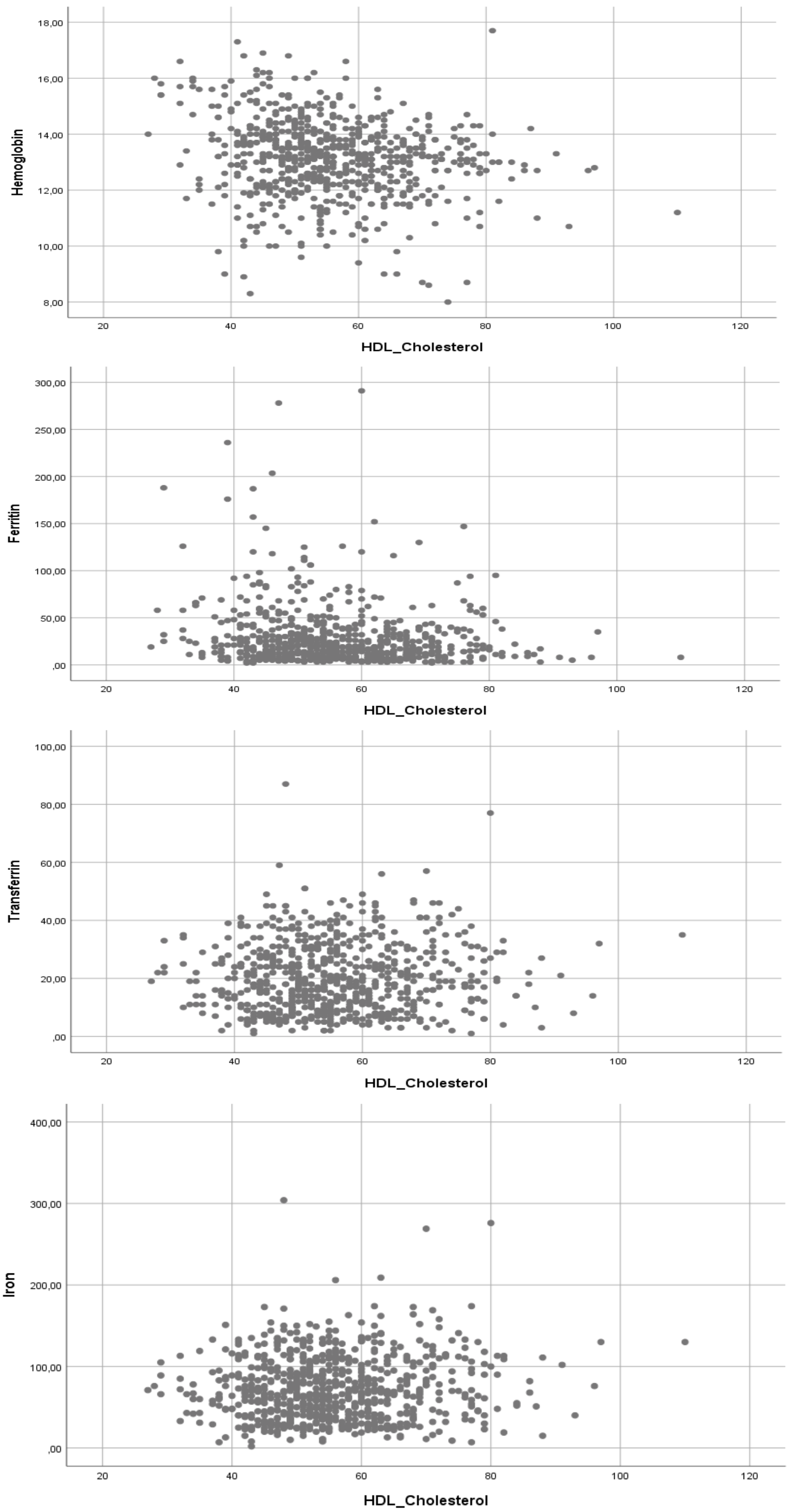

Supplementary Figure S4. **Scatter plots showing the relationship between HDL-cholesterol and blood parameters**
